# Coexisting Disorders and Problems in Preschool Children with Autism Spectrum Disorders

**DOI:** 10.1155/2013/213979

**Published:** 2013-04-23

**Authors:** Lotta Höglund Carlsson, Fritjof Norrelgen, Liselotte Kjellmer, Joakim Westerlund, Christopher Gillberg, Elisabeth Fernell

**Affiliations:** ^1^Gillberg Neuropsychiatry Centre, Sahlgrenska Academy, Gothenburg University, Kungsgatan 12, 411 19 Gothenburg, Sweden; ^2^Department of Pediatrics, Astrid Lindgren Children's Hospital, Liljeholmen, Liljeholmstorget 7, 117 94 Stockholm, Sweden; ^3^Department of Speech and Language Pathology, Karolinska University Hospital, 171 64 Stockholm, Sweden; ^4^CLINTEC, Division of Speech and Language Pathology, Karolinska Institute, 171 77 Stockholm, Sweden; ^5^Department of Psychology, Stockholm University, Frescati Hagväg 8, 114 19 Stockholm, Sweden; ^6^Skaraborgs Hospital, Department of Pediatrics, Unit of Developmental Disorders, 542 24 Mariestad, Sweden

## Abstract

*Objectives*. To analyze cooccurring disorders and problems in a representative group of 198 preschool children with autism spectrum disorders (ASD) who had had interventions at a specialized habilitation center. *Methods*. Parents and children were seen by a research team. Data were based on parental interviews, pediatric assessments, and tests of the child. Information on autistic symptoms, general cognitive function, speech and language, motor function, epilepsy, vision, hearing, activity level, behavior, and sleep was collected. *Results*. Three ASD categories were used: (1) autistic disorder (AD), (2) autistic-like condition (ALC) or Asperger syndrome, and (3) one group with autistic symptoms/traits but not entirely all its criteria met for ASD. Children with autism had a mean of 3.2 coexisting disorders or problems, the ALC/Asperger group had a mean of 1.6, and children with autistic traits had a mean of 1.6. The most common disorder/problems in the total group pertained to language problems (78%), intellectual disability (ID) (49%), below average motor function (37%), and severe hyperactivity/ADHD (33%). *Conclusions*. The results accord with the concept of early symptomatic syndromes eliciting neurodevelopmental clinical examination (ESSENCE), and highlight the need of considering ASD in a broad perspective taking also other cooccurring developmental disorders into account.

## 1. Introduction

Autism spectrum disorders (ASDs) constitute complex and heterogeneous developmental disorders, and besides the core symptoms, children with ASD display many accompanying deficits and behavioral problems. Cooccurring disorders, such as intellectual disability, attention deficit hyperactivity disorder (ADHD), language impairment, epilepsy, and various types of behavioral disorders/problems, are common and will affect daily life. To provide adequate support and intervention for the child and the family, the child's total developmental profile as well as coexisting medical disorders needs to be considered.

Increased awareness in the society of ASD, screening at child health centers (CHCs) [1, 2], and improved habilitation and intervention services have contributed to the increased prevalence rates of ASD reported now and ASD is now diagnosed in children at younger ages than previously [3].

To establish a definite diagnosis at early ages, imply some uncertainties, especially in the milder variants of the spectrum. A change of the symptom/developmental profile may occur during the child's preschool age. Among several coexisting symptoms, some may become more manifest, and some may turn out to become less prominent. The importance of having a broad view in the assessment of young children with developmental deviations has been emphasized by Gillberg [4], who has developed the concept of early symptomatic syndromes eliciting neurodevelopmental clinical examinations (ESSENCE). This concept highlights that many disorders such as attention deficit/hyperactivity disorder (ADHD), oppositional defiant disorder (ODD), tic disorders, developmental coordination disorder (DCD), language disorder, and intellectual disability (ID) may coexist in children in different combinations, and all these mentioned disorders may coexist with ASD. Many of these disorders are also accompanied by different types of behavioral problems—tantrums, sleeping problems, feeding problems, and sensory hyper- or hyposensitivities.

In young children, the dominating disorder or problem may not be clear, and there is a definite need to follow these children's developmental trajectory over time.

The importance of having such broad view was illustrated by Levy and collaborators [5] who examined cooccurring non-ASD diagnoses and symptoms in a population-based cohort of 8-year-olds with ASD, identified from 2,568 children in a multisite surveillance program. The cooccurrence of at least one other developmental diagnosis was 83%, and at least one other psychiatric diagnosis was found in 10%.

We have previously presented data from a representative group of about 200 children with ASD referred for intervention at a specialized habilitation center for young children with autism. Data on these children were collected at referral to the center and after two years during which the child had received intervention, mainly based on applied behavior analyses (ABAs) of varying intensities [6, 7].

The aim of this study was to give a comprehensive picture of these preschool children's coexisting conditions identified at the two-year follow-up and to relate these to the types of ASD.

## 2. Methods

### 2.1. Participants

The total group consisted of 198 children, 29 (15%) girls, aged from 4.5 to 6.5 years, who had received intervention for 2 years at a specialized habilitation center for young children with ASD in Stockholm. Details regarding the recruitment process are described in the previous paper [6] and data regarding the group at follow-up, after two years, in a following paper [7]. Of the 198 children, 119 (60%) had two Swedish born parents. All parents of children enrolled in the study communicated in Swedish or English. The children were assessed by our research team, consisting of two neuropediatricians, one pediatrician, and one child psychiatrist, two speech and language pathologists, and two psychologists [7].

Of the 198 children, 106 children were considered to meet criteria for autistic disorder (AD), 58 had autistic-like condition (ALC), 13 had Asperger syndrome, and 21 were considered not to meet full criteria for ASD at follow-up but had autistic traits. Four of these 21 children had an intellectual disability (ID). In the follow-up assessment, a DISCO interview was performed to ascertain the child's type of ASD, and all ASD diagnoses were based on DSM-IV criteria [7].

The group was considered to be relatively representative of ASD except for the most severely disabled children with ASD, such as those with very severe epilepsy or severe syndromes and a few other children who had their follow-up at ordinary habilitation centers, and therefore they are not included in our study [6].

### 2.2. Data Collection

A clinical interview with at least one of the parents had been carried out by one of the four physicians in the team. During the same visit, a clinical observation and physical developmental examination of the child were performed. The clinical interview followed a structured questionnaire and included a detailed developmental history and information about the child's current clinical symptoms. Moreover, the children were tested by a psychologist. Children without ID were also evaluated by an experienced speech and language pathologist.

#### 2.2.1. Intellectual Function

Each child was invited to a cognitive assessment by an experienced psychologist with Griffiths' developmental scales [8] and/or WPPSI-III [9]. Intellectual disability (ID) was defined as a total IQ < 70.

#### 2.2.2. Language

Within the project, all children without ID (*n* = 101) were invited to an assessment of receptive and expressive language carried out by a speech and language pathologist using the following tests: (1) Reynell developmental language scales III [10], (2) SPIQ [11], (3) Illinois test of psycholinguistic abilities (ITPA) [12] and (4) Processability test (grammar screening) [13]. A child was classified as having a language problem if the performance was below a set criterion in two or several of the tests. In addition, all children with ID were considered to have a language problem.

#### 2.2.3. Motor Function

Motor function was estimated according to the motor skills domain score of the Vineland Adaptive Behavior Scales (VABS) [14]. A motor function problem was considered to be present if this score was below 70, that is, corresponding to below—2SD from the mean of 100.

#### 2.2.4. Epilepsy

Epilepsy was documented when the child had a diagnosis of epilepsy confirmed in a following medical assessment [15].

#### 2.2.5. Vision and Hearing

Visual impairments were recorded when parents reported that the child had a diagnosis of visual impairment, verified by an ophthalmological examination. A hearing impairment was recorded when this was confirmed by a hearing test. Vision and hearing tests are included in the 4-year health assessment at child health centers. Children who cannot cooperate or fail in these assessments or when there is a suspicion of visual or hearing problem before this age are referred for ophthalmological and/or a hearing examination.

#### 2.2.6. Activity Regulation and Behavioral Problems

Parents were asked if the child had behavioral disorders or problems, including severe hyperactivity or diagnosed ADHD, severe hypoactivity, and problems with severe outbursts or severe sleeping problems. Activity regulation was also observed and noticed by the examining physician.

### 2.3. Data Analyses

A between-subjects ANOVA followed by post hoc tests (Fisher LSD) was used to examine if the mean number of coexisting problems differed significantly between ASD groups. An alpha level of .05 was used.

### 2.4. Ethics

The study was approved by the Ethics Committee in Stockholm.

## 3. Results

**Figure 1:**
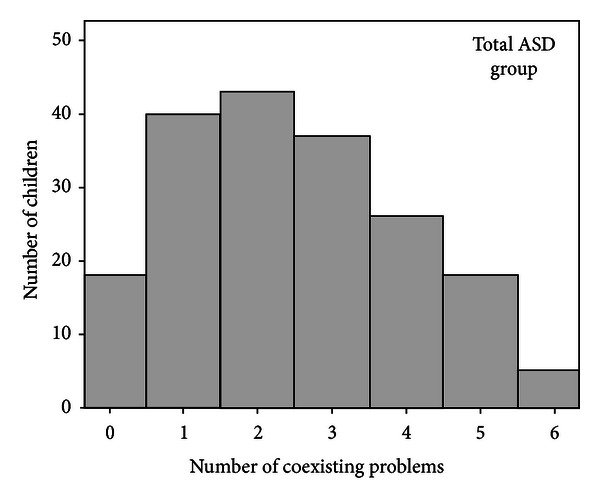
Numbers of coexisting problems/disorders in the total group of children with ASD (*n* = 198).

Of the 198 children, 181 (91%) had at least one coexisting disorder or problem, Figure 1. A between-subjects ANOVA showed that the mean number of coexisting problems differed significantly between ASD groups, *F*1,184 = 35.94, *P* < .001, *η*2_partial_ = .28.

**Figure 2:**
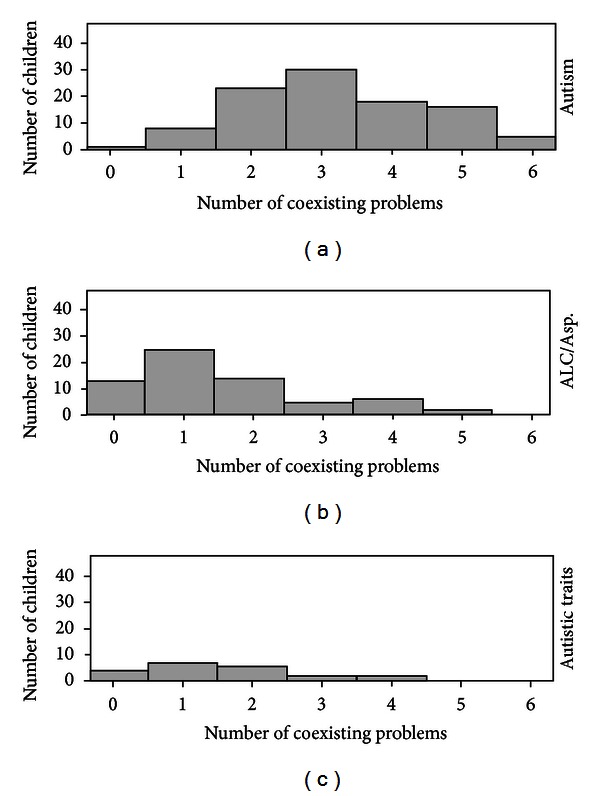
Numbers of coexisting problems/disorders in the three different ASD groups.

In the group of 106 children with AD, the mean number of coexisting disorders or problems was 3.2 (SD 1.4) (range 0–6), in the 71 children with ALC/Asperger syndrome, the mean number was 1.6 (SD 1.3) (range 0–5), and in the 21 children with autistic traits but not a full ASD diagnosis, the mean number of coexisting disorders or problems was 1.6 (SD 1.2) (range 0–4), Figure 2.

Post hoc test (Fisher LSD) revealed that the difference between the autism group and the ALC/Asperger group as well as the difference between the autism group and the Autistic traits group were significant (*P* < .001 for both comparisons). The difference between the ALC/Asperger group and the autistic traits group was not significant (*P* > .1).

### 3.1. Language

The most common recorded problem was related to receptive and expressive language. Of the 101 children without ID, parents of 94 children accepted to let their children participate in a language assessment. Of these, 94, 53 (56%) had a definite problem; that is, they fell below a set criterion in two or more of the language tests. The remaining 41 children either failed on one test only or passed all language tests.

When the children with ID (*n* = 95) and the children without ID who exhibited a definite language problem according to the assessment (*n* = 53) were included and the 7 children without ID who could not be assessed by a speech and language pathologist were excluded from the 196 children who had a DQ/IQ assessment, the rate of language problems was 78% (148/189).

### 3.2. Intellectual Disability

Of the 196 who had a cognitive test in the project, 95 (49%) received full DQ/IQ below 70. ID was more common in the group with AD, 80/105 children (75%), compared to the group with ALC/Asperger syndrome, 10/71 children (14%), and autistic traits, 4/21 children (19%).

### 3.3. Motor Function

More than a third of the children who had Vineland interview data (71/194; 37%) had a motor skills function below −2SD corresponding to a Vineland domain score below 70. Of these 71 children, 51 (72%) also had ID.

### 3.4. Activity Regulation

Severe hyperactivity or diagnosed ADHD was recorded in 63/198 children (32%) and severe hypoactivity in 6 children (3%). Of the 63 children with severe hyperactivity, 31 (49%) also had ID, and 39 (62%) also had AD.

### 3.5. Tantrums

Severe problems with tantrums were reported for 28/198 children (14%).

### 3.6. Sleeping Problems

Severe sleeping problems were reported for 24/198 children (12%).

### 3.7. Vision and Hearing

Any kind of visual impairment or strabismus was reported in 21/198 children (11%). Of these 21 children, 10 (48%) had ID. A hearing impairment was recorded in only one child (0.5%).

### 3.8. Epilepsy

At this time, 17 children (9%) had diagnosed epilepsy. Of these 17, 12 (71%) also had ID.

## 4. Discussion

Coexisting disorders and problems, in areas of many developmental and cognitive domains, including language, intellectual disability, and behavior, and with regard to motor function and epilepsy, were very common in this group of young children with ASD. When different subgroups of ASD were considered, children with AD had significantly more coexisting disorders compared to the group with autistic like condition/Asperger syndrome or those with autistic traits. The same finding with significant differences between diagnostic groups was also reported by Horowitz and collaborators [16] in their study of cooccurring psychiatric symptoms in toddlers with ASD.

The most common coexisting disorder in our study group was language problems occurring in 78% of the total group. The children comprised, on the one hand, the 95 children who due to their general cognitive impairment, ID, were considered to have a definite language problem, and on the other hand, the 53 out of the 94 children without ID (56%) who had been assessed by a speech and language pathologist and had been found to exhibit language problems. Language and/or communicative impairments or problems of different types and severities are part of the autism spectrum and vary highly according to the general intelligence of the child with ASD and according to the type and severity of the ASD per se. The previous, mentioned concept of ESSENCE highlights relationships between language delay/disorder and other developmental disorders [4]. In many children with autism, language delay is the presenting symptom that will entail further developmental evaluation. The importance of considering language delay in a wider developmental perspective was demonstrated by Miniscalco and collaborators [17] who followed children with marked language problems, as identified at the child health screening at the age of 2.5 years. At the age 7 years, 72% of the children were found to have a major neuropsychiatric or learning disorder.

Almost half of the preschool children had ID in combination with ASD. In the group with AD, the rate of ID was 80/106 (75%) which is in accordance with O'Brien and Pearson [18] who found that autism is more common among individuals with ID and increases with lower levels of IQ.

Low motor skills function was found in about a third in this study group. Motor function in children with ASD relates to the wide aetiological panorama of ASD and to the general cognitive function. In our group, 72% of those with low motor function also had ID. In our previous study, we found that age at unsupported walking in this group of children with ASD differed significantly from Swedish norms [6]. There was a clear correlation between late onset of walking and ID, present in almost all children who started to walk after the age of 18 months.

Autism and attention deficit disorder cooccur to a considerable degree. Both disorders are relatively common with ADHD showing a prevalence of 5% [19] and ASD a prevalence of about 1% [20] in the general population. Comorbidity of ADHD in ASD patients has been found to be around 30% as estimated in an epidemiologically based study [21]. The authors underlined the necessity of always evaluating cooccurring psychiatric disorders in children with ASD since these may provide targets for intervention. Evidence for overlapping genetic influences on autistic and ADHD behaviors was reported by a UK study based on a community twin sample [22]. There was a substantial overlap between ASD and ADHD; 41% of children who met criteria for ASD had suspected ADHD, and 22% with suspected ADHD met criteria for ASD. The findings support the idea that there are some common genetic influences operating across autistic traits and ADHD behaviors throughout normal variation as well as at the extreme. As pointed out in the paper by Frazier and collaborators [23], the importance of considering stimulant medication also in children with PDD/ASD and ADHD to improve adaptive behavior must be considered for this patient group.

Common challenging behaviors in children with autism are aggression, property destruction, disruptions/tantrums, impulsivity, self-injurious behaviors, and stereotypies [24]. These behaviors are the targets of many intervention programs of today for children with autism. The relatively low rate of behavioral problems/tantrums in our study group may be due to the early interventions that had been provided in this group. The applied behavior analysis (ABA) programs incorporate methods to modify and improve problem behavior [25]. The importance of the concept of an “autism-friendly environment” has been emphasized by Billstedt and collaborators [26] and conveys important considerations.

In our group, 12% of the children exhibited severe sleeping problems, mainly with insomnia. In a Norwegian study, sleep problems in children with autism were reported to be more than ten times higher compared to controls. The authors also found that the sleep problems were more persistent over time, implying a need for increased awareness of these problems in children with autism [27].

In our group of preschool children, epilepsy was found in 9%, and the rate can be expected to rise over time. The prevalence of epilepsy in samples of children with ASD varies according to the age group studied, etiology of ASD, and cooccurring ID. In our group, 71% of the children with epilepsy also had ID [15]. In the study by Bolton and collaborators [28], seizures in the majority of children with autism began after 10 years of age.

## 5. Limitations

In this study, only definite disorders or severe problems were considered. There were additional children with parental reports of some or minor problems, that is, those with borderline intellectual function and those with signs of hyperactivity but not definitely deviant from developmental age. There were also children who failed in one of the speech and language tests, but this was not considered to be a definite problem. However, in some children the problems may be more overt over the coming years. We did not have resources to examine all children with ID with regard to specific language problems. Another limitation is that we considered that all children with ID would have a language problem due to their general cognitive impairment. Other limitations are due to the lack of formal testing for ADHD and motor performance. Data on tantrums and sleeping problems were only collected through parental interview and not from parental diaries.

## 6. Conclusion

Our study of a representative group of preschool children with ASD demonstrates that most children had additional disorders or developmental problems. There were also children in this study group with minor developmental deviations which may turn out to become more significant over time. Cooccurring ID was strongly related to the presence of many other comorbidities, such as motor skills problems, hyperactivity, epilepsy, and visual problems.

The interplay of many disorders and problems cooccurring with ASD illustrates the validity of the concept of ESSENCE. About 90% of the children in our cohort of children with ASD, consisting of about 200 young children, exhibited other problems than the ASD per se. Thus, coexisting conditions should always be looked for in the assessment procedure.
